# Neural Cognition and Affective Computing on Cyber Language

**DOI:** 10.1155/2015/749326

**Published:** 2015-09-28

**Authors:** Shuang Huang, Xuan Zhou, Ke Xue, Xiqiong Wan, Zhenyi Yang, Duo Xu, Mirjana Ivanović, Xueer Yu

**Affiliations:** ^1^Overseas Training Center, Shanghai International Studies University, Shanghai 200083, China; ^2^School of Humanities and Social Science, Sichuan Conservatory of Music, Chengdu 610021, China; ^3^School of Media & Design, Shanghai Jiaotong University, Shanghai 200240, China; ^4^School of Mathematical Sciences, Fudan University, Shanghai 200433, China; ^5^School of Software, Fudan University, Shanghai 200433, China; ^6^Department of Arts Management, Tianjin Conservatory of Music, Tianjin 300171, China; ^7^Department of Mathematics and Informatics, Faculty of Sciences, University of Novi Sad, 21000 Novi Sad, Serbia; ^8^College of Arts and Science, Washington University in St. Louis, St. Louis, MO 63130, USA; ^9^Marketing Department, J.L. Kellogg School of Management, Northwestern University, Evanston, IL 60208, USA

## Abstract

Characterized by its customary symbol system and simple and vivid expression patterns, cyber language acts as not only a tool for convenient communication but also a carrier of abundant emotions and causes high attention in public opinion analysis, internet marketing, service feedback monitoring, and social emergency management. Based on our multidisciplinary research, this paper presents a classification of the emotional symbols in cyber language, analyzes the cognitive characteristics of different symbols, and puts forward a mechanism model to show the dominant neural activities in that process. Through the comparative study of Chinese, English, and Spanish, which are used by the largest population in the world, this paper discusses the expressive patterns of emotions in international cyber languages and proposes an intelligent method for affective computing on cyber language in a unified PAD (Pleasure-Arousal-Dominance) emotional space.

## 1. Introduction

In today's society, cyber space has become an important place for people to share information, exchange opinions, and communicate emotions. Due to its virtuality, autonomy, openness, inclusiveness and the high expressiveness owing to various technologies of new media, the language creativity of people has been inspired to the extreme, giving rise to the booming of cyber languages [1].

Professor Yu at Communication University of China pointed out that cyber language is a “unique natural language” commonly used in cyber space [2]. According to Ferdinand de Saussure's semiotic theory, Chinese scholars have classified cyber languages into the two categories of readable symbols and nonreadable symbols and studied their symbol system, ideographic features, and the formation rules [1, 3, 4]. However, there has not been a consistent definition of cyber language (network language, Internet language, or web language) so far. With the rapid development of modern communication and new media technologies, the Internet, the Internet of things, and the wireless communication network have been integrated into the omnipresent “ubiquitous network” which features increasingly varied expression patterns of cyber language, including icon, audio, video, and text as well as shapes, colors, and brightness. Based on all the findings from previous researches, we define cyber language as “a symbol system that people have agreed on and widely used in communication under the ubiquitous environment.”

Cyber languages are very rich in the expression of emotions by either the simple assembly of readable and nonreadable symbols or the complexes of texts, icons, audio or video signs, and their hybrids [5]. Any changes in the component, shape, color, layout, or presentation sequence may deliver different emotional messages. Cyber language is developing fast in all the world major languages such as Chinese, English, Japanese, German, French, and Spanish [1–3]. In the context of globalization, cyber language has brought new vitality to international language communication and strong capacity for expressing emotions. Therefore, research about emotional characteristics in cyber languages has attracted wide attention from linguistic study to public opinion analysis, internet marketing, service feedback monitoring, and social emergency management.

Affective computing, originally presented by Picard in 1997, indicates that emotional information can be perceived, processed, and computed by machine [6], which has been applied to cyber space in online opinion analysis [7], smart service design [8], psychological monitoring in ubiquitous learning [9], and dynamic emotion computation on vocal social media [10]. In the past decades, although great progress has been made with affective computing on natural language, there are still a lot of difficulties in dealing with cyber language due to its complexity and variability. Also, the cognition of emotional symbols in cyber language is closely related to the neural activities of human beings and affected by such factors as nationality and cultural background, which requires further multidisciplinary research on this issue.

Firstly gave a classification of emotional symbols in cyber language according to the Discovery Learning Theory and then analyzed the cognitive characteristics of different symbols. Based on our previous research findings, a mechanism model to show the dominant neural activities in that process was put forward. In order to analyze the expressive patterns of emotions in international cyber languages, a comparative study of Chinese, English, and Spanish languages was conducted in this paper, and finally an intelligent method was proposed for affective computing on the readable texts and nonreadable symbols in a unified PAD emotional space.

## 2. Emotional Symbols in Cyber Language and Neural Cognition

### 2.1. Classification of Emotional Symbols

The symbols that can be used to express emotions in cyber languages are very abundant and continuously innovative and include the simple assembly of readable and nonreadable symbols or the complexes of texts, icons, audio, video signs, and their hybrids [5].

According to the Discovery Learning Theory, the learning process is realized by the learner's cognitive representation, which refers to the mental process of turning perception of external substances into internal mental facts. The manner of cognitive mental representation will experience three stages as people grow up: first enactive representation, second iconic representation, and, third, symbolic representation, which show the sequence in human's cognition of different information types, that is, enactive information comes first and is followed by image information and then text information.

In order to study the impact of different types of information on emotional cognition in cyber languages, we classify the emotional symbols into six categories (ECSAGT) [5, 7]: enactive symbols, color symbols, structural symbols, audio symbols, graphic symbols, and text symbols, each of which delivers emotional messages by following certain encoding and commonly accepted rules.

### 2.2. Cognitive Characteristics of Emotional Symbols

The analysis of emotional symbols in cyber languages is related to the intention and expression of the information sender as well as the perception and cognition of the information receivers. The emotions of the sender and possible receivers are different, so we should determine that our target is to identify the sender's emotions from his presented symbols or to judge the activated emotions of the receivers by the information of those symbols, which will be evaluated based on the statistical significance [10]. In affective computing, we usually consider the latter.

According to researches in cognitive neuroscience, human emotions arise from the external signals, transmitted through peripheral sensory organs and the internal sensory pathways to the brain's limbic system where the rapid primary emotion is produced, followed by a relatively slow secondary emotion formed in the interaction of the higher cognitive limbic system and the cerebral cortex [11, 12]. This process is controlled by the emotional circuits of the human brain and will give rise to activation responses in corresponding brain regions.

Recent researches into human emotions have been well supported by updated experimental technologies such as EEG (electroencephalograph), ERPs (event-related potentials), fMRI (functional magnetic resonance imaging), and DTI (diffusion tensor imaging). In particular, the blood oxygenation level dependent functional magnetic resonance imaging (Bold-fMRI), with such advantages as being noninvasive, nontraumatic, and capable of locating accurately the activated brain areas, has been applied to the studies of language and emotion's neural mechanism [13–15].

In our previous research organized by Professor Dai et al. at Fudan University, we found that the cognitive responses to different types of symbols varied from one to another through experimental observation by EEG and fMRI [7]. For example, enactive, structural, color, and graphic symbols usually take less time and can give rise to the rapid primary emotions, which we call the primary emotional information. The semantic text symbols will take up more time while perceived by the advanced cortex in the brain. They usually generate the slower secondary emotions and belong to the secondary emotional information. Audio symbols contain both representational information and semantic information and therefore bear the characteristics of the primary and the secondary emotional information, but take more time resources than the first type. The primary emotional information is cross-cultural and independent of languages to a large extent. Once the cognitive rules of emotional symbols come into being, the secondary emotional information plays a vital role in expressing more in-depth emotion. The characteristics discussed above should be considered in the affective computing on the messages composed of mixed types of emotional symbols so as to reflect the dynamic cognitive responses to those symbols.

As one of the essential issues in Principles of Visual Communication (PVC), the cognitive characteristic of visual constructs has been studied for many years [16]. Furthermore, researchers and engineers in the field of human computer interaction (HCI) have developed effective computational models to measure the time characteristics of different elements in that process [17, 18].

**Figure 1:**
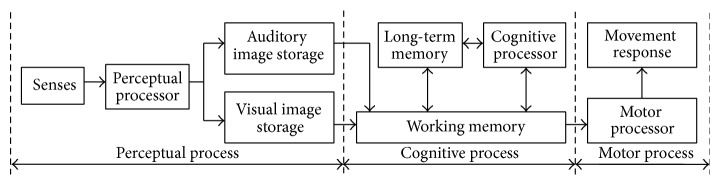
Information process in human processor model.

According to the human processor model (a.k.a. MHP) [19], the information process includes three subprocesses: Perceptual Process, Cognitive Process, and Motor Process, as shown in Figure 1. Time parameters in the Perceptual Process and Cognitive Process can be measured as in Table 1 [20].

**Figure 2:**
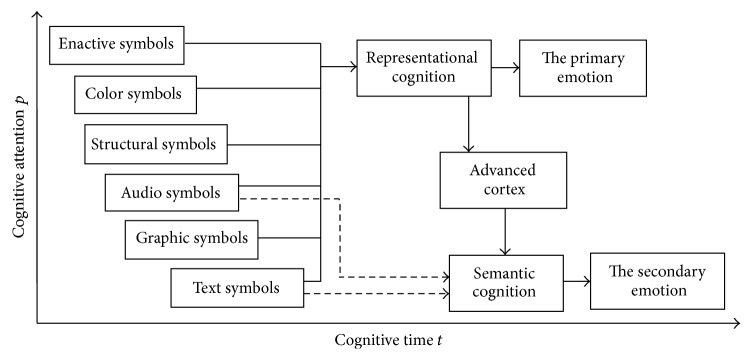
General cognitive characteristics of different types of emotional symbols in cyber language.

From Table 1, we can find that the processing time of visual information is usually shorter than that of auditory information. However, in our study, the visual information involves enactive symbols, color symbols, structural symbols, graphic symbols, and symbols, which have different processing time. If only considering the simple action, color, and structure of those symbols in cyber language, the experiment showed that the faster orders of processing time are: enactive symbols, color symbols, structural symbols, audio symbols, graphic symbols, and text symbols in representational cognition [7]. Compared with the audio symbols, graphic symbols can be perceived faster but they need more processing time to be understood in the cognitive process. However, people can usually cognize the semantic meaning of audio symbols much faster than that of text symbols. Therefore, we offer a schematic diagram as Figure 2, which shows the general cognitive characteristics of different types of emotional symbols in cyber language.

### 2.3. Neural Mechanism Model

**Figure 3:**
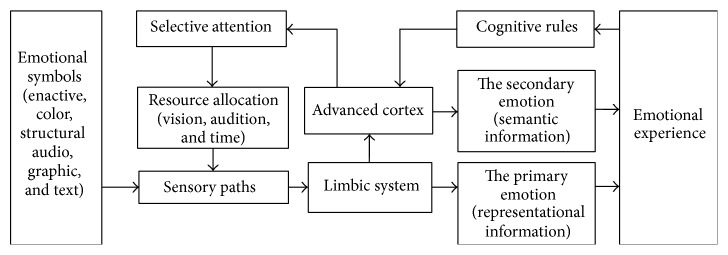
Neural cognition of emotional symbols in cyber language.

The brain mechanism of emotions has been systematically explored by scholars in the area of affective neuroscience [21–23]. In order to provide systemic guidance for analyzing the neural cognition of emotional symbols in cyber language, based on the summary analysis of existing theories and findings, we put forward a mechanism model to show the dominant neural activities in that process as in Figure 3 [5, 9].

The stimulus signals of emotional symbols will be delivered through the receiver's sensory pathways to his limbic system and produce the intuitive primary emotion and then generate the slower but more rational secondary emotion through the cognitive activities of the advanced cortex in the brain. Finally, the changes of emotion will lead to the physiological reactions which are perceived by the brain and form the specific emotional experience. Actually, the subjective assessment of an emotional symbol by the information receivers is the judgment of their emotional experiences.

In that process, the advanced cortex of the brain regulates its selective attention on the sensory pathways, that is, it distributes the visual, auditory, and time resources autonomously. The selective attention depends on the information receiver's motivation, knowledge, memory, cognition and such advanced psychological activities as decision-making. After experiencing the same symbol multiple times, a cognitive rule will be created in the memory to produce the conventional responses to familiar symbols.

## 3. Expressive Pattern of Emotions in Cyber Language

### 3.1. Corpus and Affective Vocabulary

When we look at cyber language closely, we will find that it expresses emotions with readable words or nonreadable symbols that have specific sentiment orientation. They are in general defined as emotional words, which can be found in a large quantity in world's most used languages such as Chinese, English, and Spanish. For example [24], “Bro” is a short for brother. It sounds pretty warm while “lol” expresses joy and refers to “laugh out loud” or “crack up.” In addition, there are also many special terms such as “pfffffff,” a proud word which means whatever or as you like. “Tmr” is a short for “tomorrow” and refers to something that will be done tomorrow. “N00b” is a newbie or a green hand, usually used to furiously describe those who are clumsy at certain things. “W00t” stands for who or what and is used to describe something or somebody that is exciting or surprising. In some countries, people may use figures or symbols to express their feelings. For example, “1337” represents “elite,” a person who is very competent. This number conveys feelings of surprise and joy. The number “56” means boring in English, aburrido in Spanish, and “*无聊*” in Chinese. The letter “D” is a big laugh. The symbol “:)” is a smile while “:(” means sadness.

A project carried out by the researchers of cyber language worldwide aims to build the corpus by collecting and sorting out frequently used cyber vocabulary and symbols such as General Inquirer, WordNet, and SentiwordNet. The corpus will include a large quantity of affective network terms, which are vital to the analysis of sentiment orientation of cyber language. A case in point is China's Hownet, which has collected 52,000 Chinese terms and 57,000 English words [24]. Among all the published ones, there are 219 words describing the intensity of emotions, 3,116 negative, 1,254 derogatory, 3,730 positive, 836 approbatory, and 38 propositional making a proposition. HowNet's Semantics Dictionary has also included a large collection of lexical semantic entries, each of which is composed of semantics and its description of a term. It offers guidance on how to analyze the above-mentioned affective expressions in a specific context.

The sentiment orientation affective cyber language presents is defined as sentiment polarity, which can be divided into three categories: positive, negative, and neutral. Every affective word's polarity and intensity correlates with emotional expression and cognition standards of cyber language in the specific context. The polarity and intensity of benchmark emotional words in the typical context can be identified through statistical studies of cyber language and has been covered by many corpuses, such as that of Chinese affective lexicon ontology collated and annotated by the Information Retrieval Laboratory of Dalian University of Technology. It provides thorough accounts from different perspectives of a word's part of speech, emotion category, intensity, and polarity and therefore offers important standards for the calculating of parameters related to affective cyber vocabulary.

Cyber languages are characterized by wide-ranging vocabulary that is profuse in sentiments and updated rapidly. The utilization of affective vocabulary is the basic method of the expression of emotions in cyber languages. Researchers worldwide have built the corpuses of cyber languages by collecting and sorting out frequently used vocabulary and symbols.

### 3.2. Expressive Pattern of Emotions

The emotional message of cyber language is decided not only by the affective vocabulary used in the sentence, but also by the expressive pattern in the whole sentence. Therefore, the same affective vocabulary can be completely opposite in meaning when expressed in a different pattern. For example, “When I just heard the news, I was quite upset. But after having lurked for a while, I found hikers were right about that, so just want to show up today to share my joy.” There are two affective words in this sentence, the negative “upset” and the positive “joy” together with a concession word “but.” Supposing *P* stands for the positive emotion word, *N* for the negative one and *T* for the concession, the emotion expression pattern of the above sentence can be generalized as the follows:

(1) $$\begin{array}{c} N+T+P⟹P. \end{array}$$

In cyber language, conjunctions are critical to the understanding of the emotional messages delivered in sentences and thereafter are important objects to be considered in the analysis of emotional structure and expression patters.  Table 2 has included some commonly used connectors in Chinese, English, and Spanish [24].

Of course, a complete emotional expression pattern also involves degree, negative words, and punctuation marks. For example, the Chinese words “不*很高兴*,” “不*高兴*,” and “很不*高兴*” express unhappiness of very different degrees. Punctuation marks such as “!”, “?”, “…” and emoticons, in particular, demonstrate very distinct sentiment orientations. In addition, the sequence of affective words in a sentence will make a difference. For example, the English sentence “We are exhausted now; above all we are so happy for our success.” adopts the pattern of “*N* + above  all + *P*⇒*P*”. The emotion of the whole sentence is determined by the phrase following “above all,” which is “so happy.” Without strict rules regulating cyber language expression, which is ever changing, we will have to use computers to automatically capture new entries and modify them with subjective cognition in order to build open corpuses of expressive patterns of emotions in sentences and analyze the messages delivered by them.

What remains a research issue is the emotional expression through some nonreadable symbols such as enactive symbols, audio symbols, structural symbols, color symbols, and graphic symbols. As with in text symbols, the most effective approach to affective computing on those emotional symbols is to establish an open and frequently updated knowledge library based on the ontology of nonreadable symbols, which is correlated with the cultural background, language context, and social environment and should be processed with emotional notations considering the language application environment [5, 9]. Among those symbols, the emotions in audio symbols are mainly reflected in the speed, intensity, pitch frequency, and spectral parameters of the audio signals and can be highly cross-cultural and cross languages. In an online conversation, an high accuracy rate of emotion recognition can be achieved in the way of pattern recognition without even semantic analysis [10, 25–27].

## 4. Affective Computing on Cyber Language

### 4.1. Unified PAD Emotional Space

In order to be processed by the machine, the affective characteristics of an emotional symbol in cyber language should be described quantitatively. The rudimentary description is its positive or negative polarity with quantitative intensity. In most cases, researchers propose the “six big” types of emotions: anger, disgust, fear, joy, sadness, and surprise [28], which have been widely applied to the analysis of graphic, audio, and video signals. However, the emotional symbols in cyber language usually carry mixed affective characteristics and reflect dynamic changes in audio and video signals.

Mehrabian presented a 3D model which can describe any kinds of complicated emotions in a PAD emotional space [29, 30]. It includes three nearly independent continuous dimensions: Pleasure-Displeasure (P), Arousal-Nonarousal (A), and Dominance-Submissiveness (D). Experiment shows that all the known emotion states can be almost described in this space very well [10, 30]. So far, the PAD model has been successfully applied in a variety of areas, such as audio-visual speech synthesis [31], micro-blog sentiment analysis [32], and music emotion comparison [33]. The 3D dimensions of PAD provide a unified space for describing the mixed affective characteristics of all types of emotional symbols in cyber language as well as their dynamic changes. As well, any emotional state in the PAD space can also be described as the percentage rates of typical emotions based on a conversion metric function [10, 34].

Usually, the PAD values of commonly used emotional symbols should be firstly evaluated by subjective assessment according to the perception and cognition of the typical information receivers in the process of emotional notation and stored in the knowledge library based on the ontology, therefore providing the references for comprehensive computation by machine. In order to achieve precise and consistent results in subjective evaluation, Mehrabian designed an initial 34-item test questionnaire [29]. However, the application in practice indicates that the questionnaire should be designed according to the specific language due to the differences in language understanding and cultural backgrounds.  Table 3 shows the Chinese version of the simplified 12-item questionnaire which was presented by the scholars from the Psychological Institute, Chinese Academy of Sciences [35]. The assessment is based on what kind of feeling is more intense in each item. From the left to the right, it is scaled within the range from “−4” to “4.” The scores in each item may be calculated and converted into the normalized values of P, A, and D [35].

### 4.2. Knowledge Library and Emotional Notation

**Figure 4:**
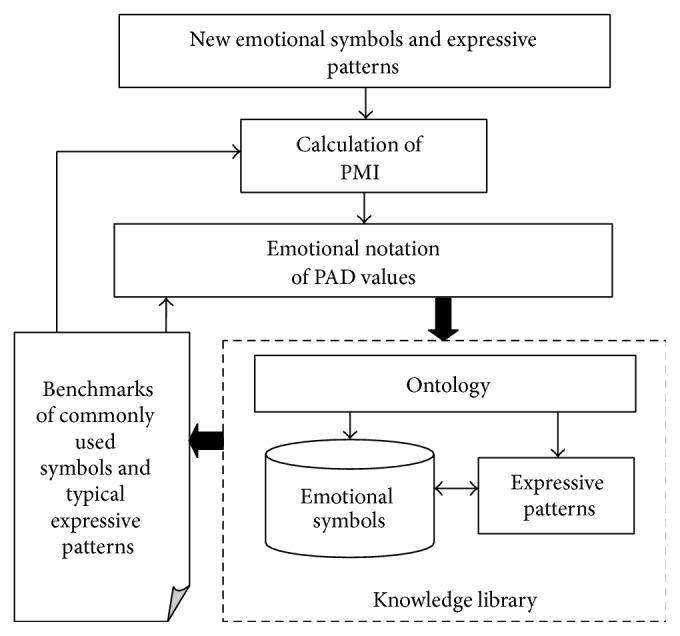
Knowledge library and emotional notation.

The perception and cognition of emotional symbols in cyber languages are not only related to neural cognition but also affected by the information receivers' background and features. Therefore, we establish an open and frequently updated knowledge library as shown in Figure 4.

The emotional symbols as well as their expressive patterns are stored in this library based on the ontology. The commonly used symbols in the library should be firstly assigned with the emotional notation of PAD values as the benchmarks through the subjective assessment which has been discussed before in this paper.

In order to evaluate the values of the rest or a new emotional symbol, we adopted the PMI (Pointwise Mutual Information) method [36], which is based on the probabilities of the new symbol and its benchmarks in the knowledge library. For example, the improved HowNet-PMI algorithm has been successfully applied to affective computing on Chinese cyber language [37].

**Figure 5:**
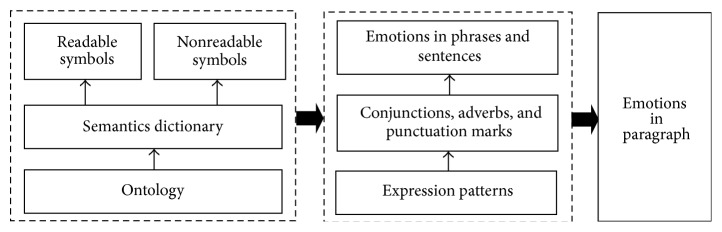
Semantic dictionary and expressive patterns in knowledge library.

As shown in Figure 5, in the knowledge library, we build up a semantic dictionary based on the ontology of international cyber languages, which includes both the readable and nonreadable symbols. The expressive patterns of emotions in sentences are represented by the knowledge such as the templates and rules which describe the commonly used structures along with the conjunctions, adverbs, and punctuation marks.

Based on the knowledge, affective computing can be carried out on a whole message which may contain one or more sentences with the additional nonreadable symbols.

### 4.3. Intelligent Computing Method

**Figure 6:**
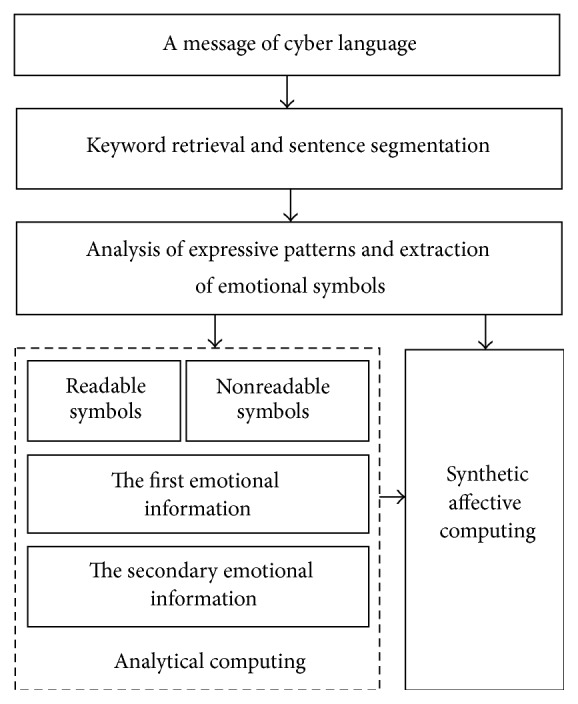
Basic process of affective computing on cyber language.

The basic process of affective computing on cyber language is shown as in Figure 6. It includes the following four steps [5]:

1. The first step is Keyword Retrieval and Sentence Segmentation. A message of cyber language will be segmented into one or more sentences with possible additional nonreadable symbols for further processing by the structure analysis. Chinese, English, and Spanish have their different patterns in sentences, which can be mostly structured by the retrieved keywords such as conjunctions, adverbs, and punctuation marks.
2. The second step is Analysis of Expressive Patterns and Extraction of Emotional Symbols. In this step, each segmented sentence needs to be broken up into a series of separate words by dedicated tools such as the Chinese word segmentation system NLPIR [38]. Hereafter, the expressive patterns of emotions in sentences with additional nonreadable symbols are analyzed, and all emotional symbols in the message are extracted based on the semantics dictionary, structural templates, and rules which are stored in the knowledge library.
3. The third step is Analytical Computing. In this step, the readable and nonreadable emotional symbols are computed separately. In order to reflect the dynamic affective features in human cognitive processes, the enactive symbols such as flashing signs and video signs, as well as structural, color, and graphic symbols, are computed and ranged into the primary emotional information. The semantic text symbols are computed into the secondary emotional information. Audio symbols contained both the, especially, as discussed before in this paper, it contained the representational information and the semantic information. The former is related to vocal emotion only and can be computed by the LS-SRV estimator, which has been successfully applied to Wechat and QQ [10]. The later should be firstly converted into text sentences by a speech recognition tool and is afterwards computed similarly to the text symbols.
4. The final step is Synthetic Affective Computing. The results by the step of Analytical Computing will hereafter be synthesized and adjusted based on the analysis of conventional expressive patterns, in order to reach a more accurate and comprehensive result. Dynamic affective features in the message of cyber language may be represented by the primary and the secondary emotions as well as the changing positions in a text sentence.

**Figure 7:**
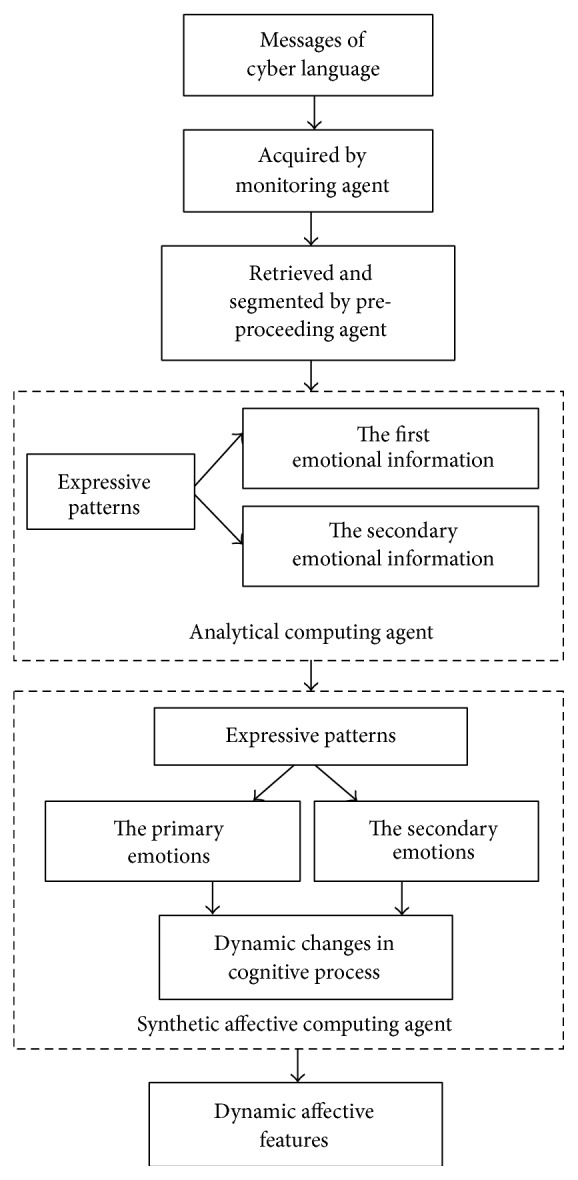
Intelligent computing method.

This process in Figure 6 can be implemented by the intelligent computing method as shown in Figure 7.

In the proposed method, the processing and computing tasks are accomplished by a multiagent system intelligently. It includes a Monitoring Agent, a Preprocessing Agent, an Analytical Computing Agent, and a Synthetic Affective Computing Agent. Different from the traditional computing on emotions, considering the characteristics of neural cognition, our method can give the results of the primary and the secondary emotions, respectively, and shows their dynamic changes. This is significant for the further analysis of emotion propagation through social media in cyber space [39–41].

## 5. Experiment and Result

**Figure 8:**
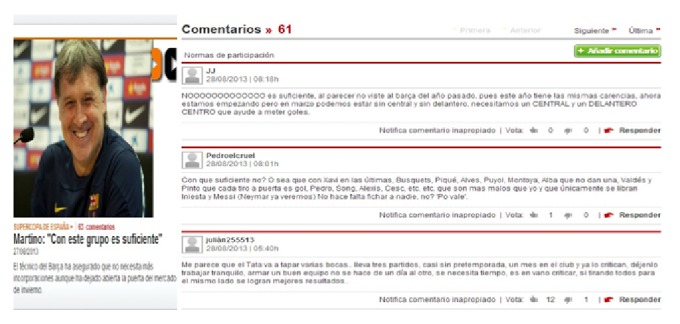
Sports news on* Mundo Deportivo* and the readers' online comments.

**Figure 9:**
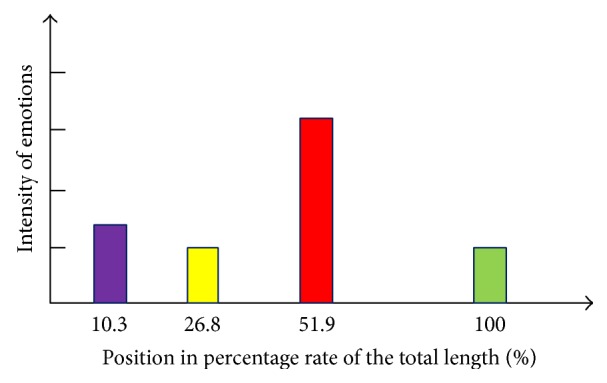
Dynamic changes of affective features in the online comments.

Figure 8 is a piece of sports news reported by the famous Spanish newspaper* Mundo Deportivo* and the readers' online comments. There are total 72 comments on this news [24]. Once seeing this footage, the audiences are firstly attracted by the smiling pictures and videos of the news reporter and produce the primary joy emotion immediately the primary emotion. After computing on the online comments, we get the main types of emotions in different positions of the total comment text as shown in Figure 9, which reflect the dynamic changes of the secondary emotion in the online comments.

**Figure 10:**
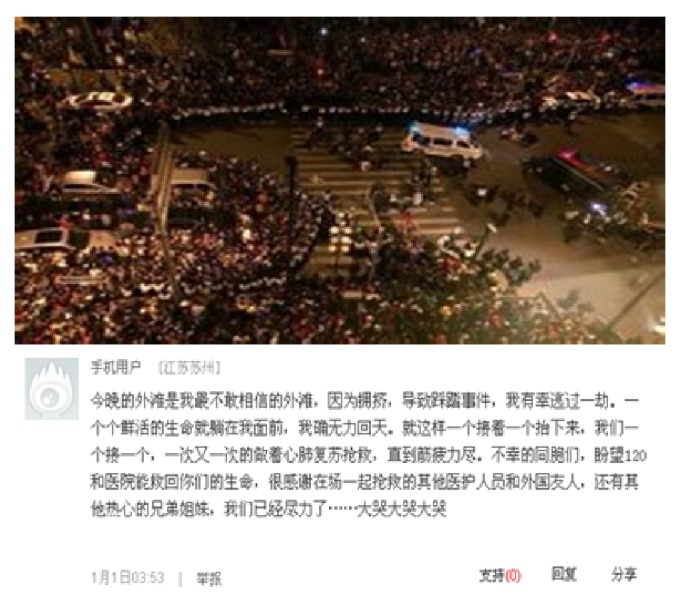
Feeling description by a survivor in Shanghai bund stampede.

Figure 10 is a feeling description posted by a Chinese survivor on January 1, 2015, at 3:53, who escaped from the miserable Shanghai bund stampede which took place at 23:35 on December 31, 2014, and resulted in 36 deaths and 49 injuries. The survivor said: “Tonight's Bund was nothing I could've imagined because of the crowding and trampling accident. I was fortunate to have survived. I saw young lives perished in front of me, but I couldn't save them. They were put on stretchers and sent down to us one after another. We tried CPR for all, one, two, three… until we were all exhausted. Poor people, hopefully 120 and the hospital can do a better job. Have to thank the medical workers, foreigners as well as all the others that participated in the rescue efforts. We have tried our best … (crying out loud).”

**Figure 11:**
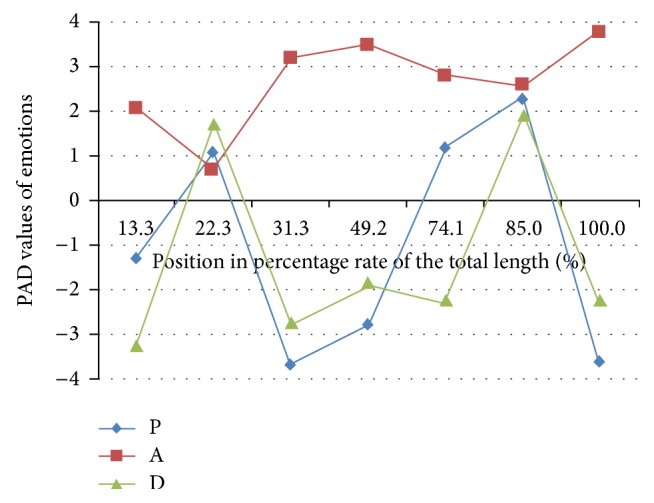
PAD values of affective computing result.

Figure 11 shows the PAD values of affective computing result on that description. It represents the strong intensity of the mixed emotions as well as their changes which include grief, despair, helplessness, and the gratefulness to the people who were involved in this rescue.

## 6. Conclusion and Discussion

With the rapid development and wide application of the Internet and ubiquitous networks, cyber space has provided people with a new virtual society and convenient living and working platform. Characterized by its customary symbol system and vivid expression patterns, cyber language not only acts as the tool for people to communicate in the cyber space, but also plays a vital role in affective exchange and emotional propagation as well as social psychology and behaviors and has caused high attention in many areas in recent years.

Due to the open, virtual, and dynamic language environment, affective computing on cyber language requires a systemic and interdisciplinary research. This paper presented a classification of the emotional symbols in cyber language and put forward a mechanism model to show the dominant neural activities in the cognitive process. Furthermore, after analyzing the expressive patterns of emotions in the languages of Chinese, English, and Spanish, this paper proposed an intelligent method for affective computing on cyber language in a unified PAD emotional space, which can deal with the multi symbol information and mixed emotions in a cyber message and show their dynamic changes according to the characteristics of the neural cognition process. Experimental results indicate that this method can reach an accuracy of more than 70% for the computing on text symbols and audio symbols and provide an effective approach to the application of a lot of areas such as public opinion analysis, internet marketing, service feedback monitoring, and social emergency management. However, the processing of the remaining nonreadable symbols had to be made by subjective evaluation in most cases.

In the future, the language ecosystem of cyber language and new media technologies will be ever changing and continuously updated. We suggest that future studies can be conducted in the following areas: (1) How to build an open and dynamic updated knowledge library to assist the affective computing by applying intelligent monitoring and big data mining techniques; (2) how to establish more thoroughly expressive emotional patterns and provide statistical fundamental parameters for the elaborate description of neural cognition by using advanced experimental observation techniques; and (3) how to explore a more effective method for computing on nonreadable symbols such as enactive and structural symbols.

## Figures and Tables

**Table 1:** Time parameters in the Perceptual Process and Cognitive Process.

Parameter	Mean	Range
Decay half-life of visual image storage	200 ms	90–1000 ms
Decay half-life of auditory storage	1500 ms	90–3500 ms
Perceptual processor cycle time	100 ms	50–200 ms
Decay half-life of working memory	7 sec	5–226 sec
Cognitive processor cycle time	70 ms	25–170 ms

**Table 2:** Commonly used connectors in Chinese, English, and Spanish.

Functions of connectors	Chinese	English	Spanish
Alternatives	① 不是…就是…	① Either…or…	① ni…ni…
	② 即不是…也不是…	② Neither…nor…	② no…tampoco…
	③ *或者*	③ Or	③ y
	④ 以及	④ As well as	④ tambien
	⑤ 和, 与, *并且*	⑤ And	⑤ y
	⑥ 和…都	⑥ Both…and…	⑥ ambos…y…

Cause and effect	① *因此*	① Therefore	① por lo tanto
	② 所以	② So	② así que
	③ *总之*, *因此*	③ As a result	③ por consiguiente
	④ 由于	④ Because of	④ por
	⑤ 基于, 由于	⑤ Due to	⑤ gracias a
	⑥ *因为*	⑥ Because	⑥ es que

Concession	① 还未, *仍然没*有	① Yet	① aún
	② 但是, 但	② But	② pero
	③ 而, *正当*	③ While	③ sino
	④ 相反地说, 而是	④ On the contrary	④ por el contrario
	⑤ 可是, 不过, 然而	⑤ However	⑤ sin embargo
	⑥ 然而	⑥ At the same time	⑥ mientras

Conclusion/summary	① *总之*	① In a word	① en fin
	② 整体上, 大体	② On the whole	② en general
	③ *简单*地说, *简言之*	③ In brief	③ cortar el rollo
	④ 总结下就是说	④ To conclude	④ para concluir
	⑤ *总共*, *总计*	⑤ In all	⑤ en total
	⑥ *概括*下说	⑥ To sum up	⑥ resumir

Examples	① *举例来说*	① For example	① por ejemplo
	② *在那*个*案例*上	② In that case	② en eso caso
	③ *解释*下, 说明下	③ To illustrate	③ por ilustrar
	④ 一方面地讲	④ For one thing	④ por una parte
	⑤ *打比*方说, *比如说*	⑤ Such as	⑤ tal como
	⑥ *比如*, *譬如*	⑥ For instance	⑥ entre ellos

**Table 3:** Chinese version of the questionnaire.

Question	Emotion	−4	−3	−2	−1	0	1	2	3	4	Emotion
Q1	Angry										Activated
Q2	Wide-awake										Sleepy
⋮	⋮										⋮
Q12	Influential										Influenced

Q1: angry-activated; Q2: wide-awake-sleepy; Q3: controlled-controlling; Q4: friendly-scornful; Q5: calm-excited; Q6: dominant-submissive; Q7: cruel-joyful; Q8: interested-relaxed; Q9: guided-autonomous; Q10: excited-enraged; Q11: relaxed-hopeful; Q12: influential-influenced.
